# A case of HER2‐positive male occult breast carcinoma with skin and lymph node metastases that exhibited complete response to trastuzumab monotherapy

**DOI:** 10.1002/ccr3.884

**Published:** 2017-03-17

**Authors:** Kouichi Kuninaka, Ryo Takahashi, Yutaka Nakagawa, Tadashi Nishimaki

**Affiliations:** ^1^ Division of Digestive and General SurgeryRyukyu University Graduate School of MedicineOkinawaJapan

**Keywords:** HER2, male occult breast cancer, trastuzumab

## Abstract

Metastatic male occult HER2‐positive breast cancer can be successfully treated with trastuzumab monotherapy.

## Introduction

There are only a few reports of male occult breast cancer (MOBC) 1, 2, 3, 4, 5, 6, and only two cases were positive for HER2 2, 6. One patient received trastuzumab‐containing chemotherapy in the neoadjuvant setting 6, although this did not provide a favorable outcome. Nevertheless, some reports have described the successful use of trastuzumab‐containing chemotherapy for advanced male breast cancer 7, 8, 9, and this treatment is an established and required therapy for advanced or recurrent female breast cancer 10. Therefore, we present an extremely rare case of advanced HER2‐positive MOBC that exhibited complete response to trastuzumab monotherapy.

## Case Report

**Figure 1:**
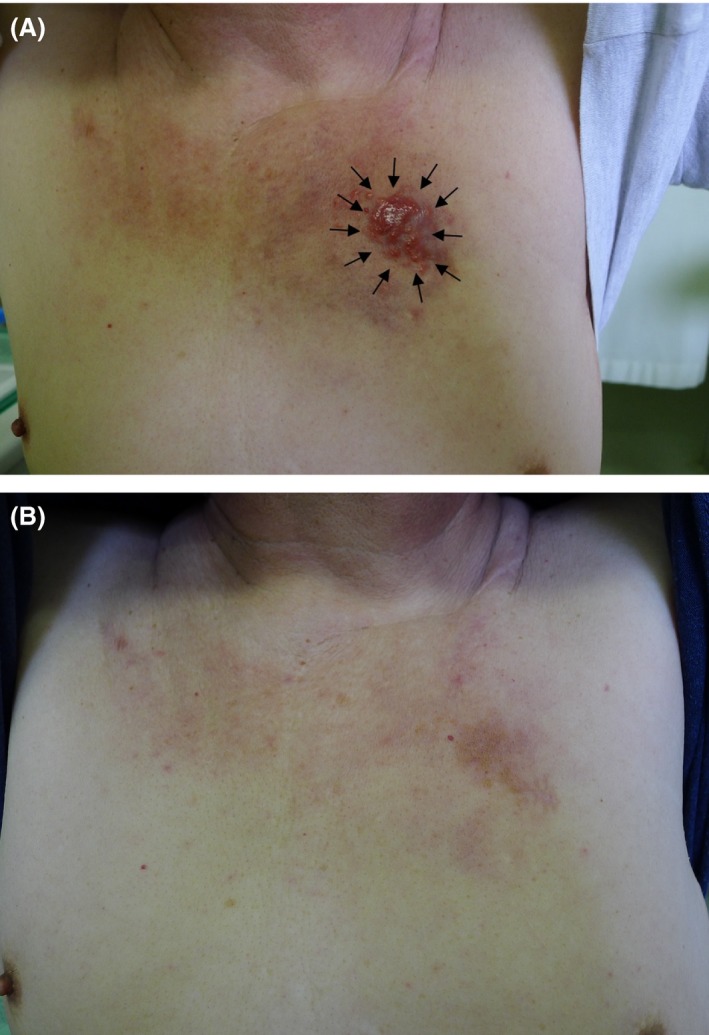
Images of the patient's chest. (A) An eczema‐like lesion was visible on the patient's left anterior chest before he received trastuzumab (arrows). (B) The patient exhibited complete response to trastuzumab monotherapy after 5 months.

A 67‐year‐old man was initially diagnosed with occult lung cancer with skin and lymph node metastases. He had been treated with four cycles of carboplatin–paclitaxel regimen and fourteen cycles of tri‐weekly docetaxel regimen which both failed to control the patient's disease. In March 2013, he was referred to our division based on computed tomography (CT) findings that elicited a suspicion of male breast cancer. Our physical examination revealed an eczema‐like reddish lesion and subcutaneous tumor (approximately 3 cm in diameter) over the left anterior chest wall (Fig. 1A). In addition, CT revealed multiple subcutaneous nodules in the left chest and multiple swollen lymph nodes in the right axilla and neck. Incisional skin biopsy was performed for the chest lesion, and the pathological findings revealed metastatic adenocarcinoma. Immunohistochemistry revealed that the cancer had a HER2 score of 3+ and was estrogen receptor (ER)‐positive, partially positive (<1%) for progesterone receptor (PR), cytokeratin 7‐positive, cytokeratin 20‐negative, and gross cystic disease fluid protein (GCDFP)15‐positive. Therefore, we diagnosed the patient with occult breast carcinoma, based on the National Comprehensive Cancer Network (NCCN) guidelines 11.

**Figure 2:**
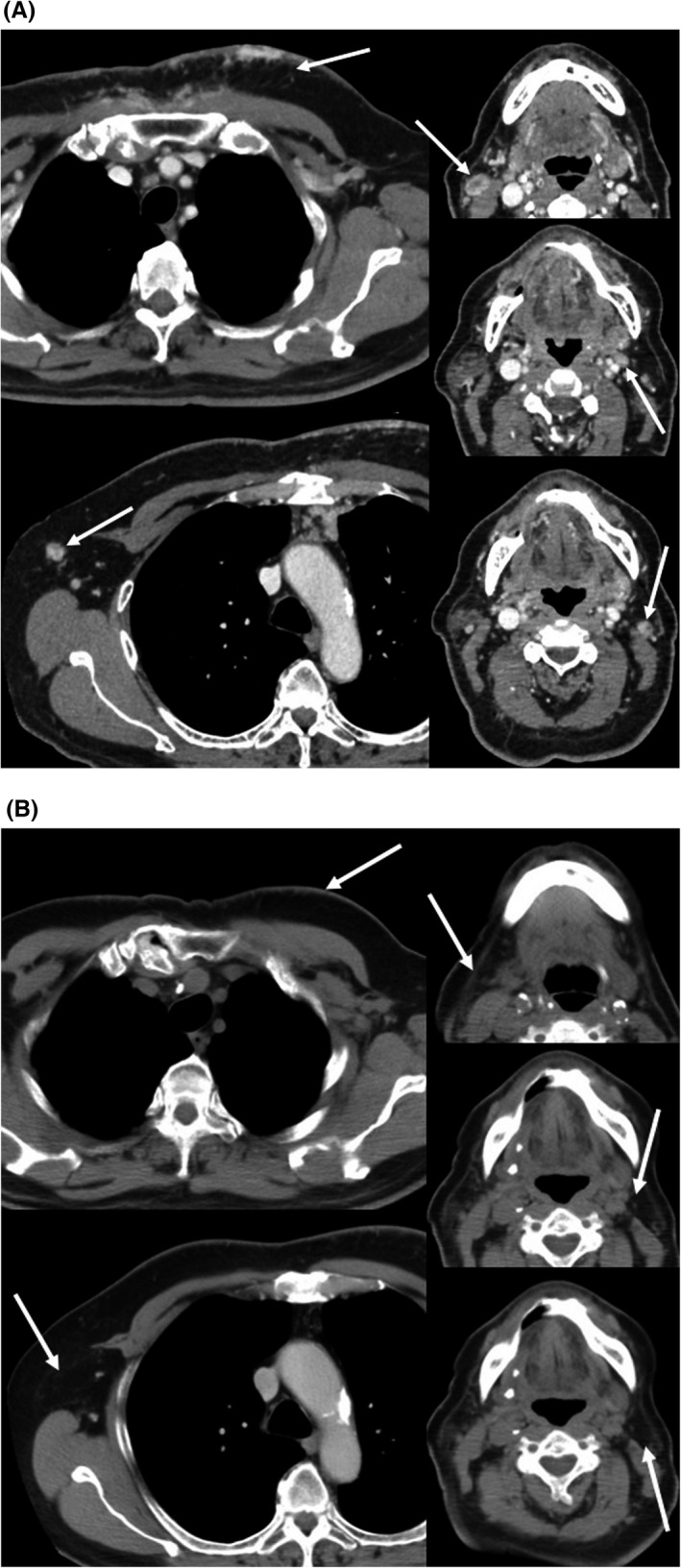
Computed tomography findings. (A) Multiple subcutaneous nodules are visible in the chest (arrow, left upper), axillary lymph node (arrow, left lower), and cervical lymph nodes (arrows, right upper, middle and lower) before he received trastuzumab. (B). The patient exhibited complete response to trastuzumab monotherapy after 5 months (arrows).

As there were no life‐threatening lesions, and the patient wanted to avoid the side effects of chemotherapy, we chose to treat him using weekly trastuzumab monotherapy (4 mg/kg for the first week and 2 mg/kg thereafter). After 5 months, the anterior chest skin lesion had completely vanished (Fig. 1B), and follow‐up CT revealed that the subcutaneous chest lesion and swollen lymph nodes had also disappeared (Fig. 2B). Therefore, we continued trastuzumab monotherapy at 6 mg/kg every 3 weeks. At 18 months after starting the trastuzumab monotherapy, follow‐up CT and positron emission tomography revealed no evidence of recurrence, and the patient elected to cease treatment. We have carefully observed the patient since that time, and CT at 15 months after cessation revealed no signs of recurrence. No obvious side effects were noted during or after the trastuzumab monotherapy, the patient reported a good quality of life during the therapy, and is now enjoying his life free from disease.

## Discussion

Male occult breast cancer is extremely rare, and only a few reports are available 1, 2, 3, 4, 5, 6. Based on the initial diagnosis of occult lung cancer, and the National Comprehensive Cancer Network guidelines for occult primary carcinoma 11, we evaluated the present patient using a skin biopsy and immunohistochemical testing for cytokeratin 7, cytokeratin 20, GCDFP, ER, PR, and HER2. The result revealed a high probability of occult breast carcinoma, and the efficacy of the trastuzumab monotherapy supports the accuracy of this diagnosis. Interestingly, none of the previous reports described testing for cytokeratin 7, cytokeratin 20, and GCDFP. Therefore, given the importance of an accurate diagnosis for guiding treatment, the present case highlights the importance of carefully complying with the relevant diagnostic guidelines.

There are two reported cases of HER2‐positive MOBC 2, 6, and one of those patients did not respond favorably to trastuzumab‐containing chemotherapy in the neoadjuvant setting 6. Nevertheless, several reports have described the successful use of trastuzumab‐containing chemotherapy for advanced male breast cancer 7, 8, 9, and one report described using trastuzumab monotherapy for maintenance after trastuzumab plus paclitaxel. In the present case, we suggested and selected trastuzumab monotherapy based on the patient's preference to avoid the side effects of chemotherapy and the tumor's clinically indolent status. We speculate that this treatment was effective because the patient's malignant lesions consisted of relatively monoclonal cancer cells that strongly expressed the HER2 protein. Furthermore, the response may be related to the sequence of treatment (i.e., chemotherapy followed by targeted therapy).

In conclusion, our findings indicate that trastuzumab may play an important role in treating cases of HER2‐positive MOBC and that trastuzumab monotherapy is a reasonable treatment option in non‐life‐threatening cases.

## Authorship

KK: corresponding author. RT: figure making. YN: co‐author. TN: administer.

## Conflicts of Interest

The authors report no conflicts of interest.

## References

[1] Namba, N. , A. Hiraki , M. Tabata , K. Kiura , H. Ueoka , T. Yoshino , et al. 2002 Axillary metastasis as the first manifestation of occult breast cancer in a man: a case report. Anticancer Res. 22:3611–3613. 12552964

[2] Gu, G. L. , S. L. Wang , X. M. Wei , L. Ren , and F. X. Zou . 2009 Axillary metastasis as the first manifestation of occult breast cancer in a male patient. Breast Care (Basel) 4:43–45. 20877684 10.1159/000193032 PMC2942011

[3] Hirao, A. , N. Oiso , J. Tsurutani , M. Kimura , M. Watatani , K. Nakagawa , et al. 2011 Transient effectiveness of an oral 5‐fluorouracil derivative, S‐1, for epirubicin, cyclophosphamide and paclitaxel refractory skin metastasis from possible occult breast cancer in a male. Case Rep. Dermatol. 3:42–48. 21487460 10.1159/000325069 PMC3073752

[4] Gonzalez‐Perez, L. M. , P. Infante‐Cossio , S. Crespo‐Torres , and F. Sanchez‐Gallego . 2012 Mandibular metastases as first clinical sign of an occult male breast cancer. Int. J. Oral Maxillofac. Surg. 41:1211–1214. 22446070 10.1016/j.ijom.2012.02.018

[5] Hur, S. M. , D. H. Cho , S. K. Lee , M. Y. Choi , S. Y. Bae , M. Y. Koo , et al. 2012 Occult breast cancers manifesting as axillary lymph node metastasis in men: a two‐case report. J. Breast Cancer 15:359–363. 23091551 10.4048/jbc.2012.15.3.359 PMC3468792

[6] He, M. , H. Liu , and Y. Jiang . 2015 A case report of male occult breast cancer first manifesting as axillary lymph node metastasis with part of metastatic mucinous carcinoma. Medicine (Baltimore) 94:e1038. 26107674 10.1097/MD.0000000000001038 PMC4504636

[7] Rudlowski, C. , W. Rath , A. J. Becker , O. D. Wiestler , and R. Buttner . 2001 Trastuzumab and breast cancer. N. Engl. J. Med. 345:997–998. 11575298

[8] Carmona‐Bayonas, A. 2007 Potential benefit of maintenance trastuzumab and anastrozole therapy in male advanced breast cancer. Breast 16:323–325. 17292609 10.1016/j.breast.2006.12.010

[9] Hayashi, H. , M. Kimura , N. Yoshimoto , M. Tsuzuki , N. Tsunoda , T. Fujita , et al. 2009 A case of HER2‐positive male breast cancer with lung metastases showing good response to trastuzumab and paclitaxel treatment. Breast Cancer 16:136–140. 18548321 10.1007/s12282-008-0060-1

[10] Callahan, R. , and S. Hurvitz . 2011 Human epidermal growth factor receptor‐2‐positive breast cancer: current management of early, advanced, and recurrent disease. Curr. Opin. Obstet. Gynecol. 23:37–43. 21500375 10.1097/gco.0b013e3283414e87 PMC4307801

[11] National Comprehensive Cancer Network . NCCN guidelines. Available at: http://www.nccn.org (accessed 25 March 2013).

